# Occurrence of mental health challenges and their association with autistic traits, cognitive level and adaptive functioning in autistic preschool children

**DOI:** 10.1192/bjo.2025.10966

**Published:** 2026-02-16

**Authors:** Weng Tong Wu, James Rufus John, Antonio Mendoza Diaz, Valsamma Eapen

**Affiliations:** School of Clinical Medicine, Faculty of Medicine and Health, University of New South Wales, Sydney, New South Wales, Australia; Ingham Institute of Applied Medical Research, Liverpool, New South Wales, Australia; Academic Unit of Infant, Child and Adolescent Psychiatry Services, South Western Sydney Local Health District, Liverpool, New South Wales, Australia; Tasmanian Centre for Mental Health Service Innovation, Tasmanian Health Service, Hobart, Tasmania, Australia

**Keywords:** Autistic spectrum disorders, child and adolescent psychiatry, evidence-based mental health, neurodevelopmental disorders, cognitive neuroscience

## Abstract

**Background:**

Mental health challenges are common in autistic individuals but there is limited research, particularly among preschool children.

**Aims:**

To ascertain the nature and occurrence of mental health challenges in autistic preschool children, as well as their association with autistic traits and cognitive and adaptive functioning.

**Method:**

We conducted a secondary analysis of data collected from children attending Autism Specific Early Learning and Care Centres across six states in Australia. The primary outcome of mental health challenges was assessed using the Child Behaviour Checklist (CBCL). The severity of autism and autistic traits, such as social communication differences and repetitive behaviours, alongside cognitive and adaptive functioning, were used as exposure variables. Multivariable linear regression analyses examined the associations among mental health challenges, autistic traits, cognitive level and adaptive functioning, and adjusted for key sociodemographic covariates.

**Results:**

Among 760 children, about 76% scored above the clinical range of CBCL total problem scores. Mental health difficulties were significantly associated with greater severity of autistic traits, social communication differences and repetitive behaviours, and lower verbal developmental functioning and adaptive functioning. Additionally, sociodemographic determinants, such as children who were older, female or with an autistic sibling, were associated with higher risk of mental health difficulties, whereas culturally and linguistically diverse status, higher parental education and family income were protective against mental health challenges.

**Conclusions:**

Our findings provide useful insights into the high prevalence of mental health difficulties among autistic preschool children, highlighting the significant association with autistic traits, cognitive and adaptive functioning levels and sociodemographic risk factors.

Autism spectrum disorder (hereafter autism) is a neurodevelopmental condition characterised by social communication differences and restricted/repetitive behaviours that begin in early childhood and persist over time.^1^ In addition to core autistic traits, autistic children may often present with mental health difficulties, including internalising and externalising behaviours at preschool age.^2^ Externalising behaviours, including irritability, aggression, self-injurious behaviour or temper tantrums, might be a presentation of frustration arising from the difficulties in expressing one’s needs.^3^ Studies have reported that autistic preschoolers and children often exhibit clinically significant aggression towards either a caregiver or another person.^4^ Autistic children might also have conflicts with colleagues or authority figures due to psychosocial and confrontation skill differences.^5^ Because irritability and other behaviours of concern commonly co-occur in autistic individuals, and these behaviours may jeopardise educational and recreational activities and even lead to in-patient psychiatric hospitalisation or residential placements, it is crucial to address and prevent these adverse behaviours with early intervention and supports.^6^

Studies have shown that internalisation of symptoms is common in autistic preschoolers, with anxiety and depression being the most frequent presentations.^2^ A study by Lugnegård et al^7^ reported that autistic young adults may be more vulnerable to anxiety because they are more aware of their differences in interpreting social cues. Similarly, autistic adults with more subtle social challenges may be at increasing risk of developing depressive symptoms.^8^ There is also evidence to suggest higher rates of withdrawn/depressed, specific phobias and separation anxiety among autistic preschoolers compared with non-autistic preschoolers.^9^ Because these internalising behaviours may increase the severity of repetitive behaviours, social communication differences and sensory deficits, further research into appropriate strategies to address these features deserves greater attention.

Because mental health difficulties commonly co-occur in autistic individuals, and these behaviours may impact access and participation in educational and recreational activities, it is important to support such issues with early interventions and support.^10^ While there is research demonstrating a link between autism diagnosis and mental health difficulties, these studies were mostly carried out in school-aged children and adolescents.^2^ Additionally, other studies^11,12^ that have examined this association among preschool populations were of small sample size, which may not accurately represent the wider population, making it difficult to draw reliable or generalisable conclusions. Furthermore, there is also a scarcity of reports exploring these associations with a social determinant of health lens that also takes into account critical psychosocial factors. To address this knowledge gap, this study examined mental health difficulties identified using the Child Behaviour Checklist (CBCL), and their association with core autistic traits, cognitive level and adaptive functioning alongside key sociodemographic factors, among a large sample of autistic preschoolers in Australia.

## Method

### Ethical standards

The authors assert that all procedures contributing to this work comply with the ethical standards of the relevant national and institutional committees on human experimentation, and with the Helsinki Declaration of 1975 as revised in 2013. All procedures involving human subjects/patients were approved by the University of New South Wales Institutional Human Research Ethics Committee (no. HC14267).

### Study design, setting, recruitment, participants and consent

Study findings are reported following the Strengthening the Reporting of Observational Studies in Epidemiology guidelines.^13^ The study is a secondary data analysis utilising data from six Australian Autism Specific Early Learning and Care Centres (ASELCCs) as part of the Cooperative Research Centre for Living with Autism (Autism CRC) ‘Autism Subtyping Project’.^14^ All children in the ASELCCs that met the criteria for autism diagnosis based on either DSM-IV or DSM-5,^1^ or had features consistent with an autism diagnosis, were invited to participate in the study. No other inclusion or exclusion criteria, nor pre-screening measures, were applied. Informed consent was obtained from all subjects and/or their legal guardian(s) for all procedures performed.

Families from each centre that were interested in the study were provided with the same set of questionnaires to complete; arrangement of time required to complete researcher-administered assessments was undertaken by either professional staff at each ASELCC or a research assistant who had been trained to research reliability. Relevant staff also attended formal training to administer the Autism Diagnostic Observation Schedule – Second Edition (ADOS-2). Further details about the intervention and study procedures are detailed elsewhere.^14^

### Study measures

#### CBCL

CBCL 1.5–5 is a self-reported questionnaire, for parents of children aged 1.5–5 years, that consists of 100 behavioural items designed to assess emotional and behavioural issues.^15^ It comprises eight subscales that can be classified into (a) emotionally reactive, (b) anxious/depressed, (c) somatic complaints and (d) withdrawn syndromes (a–d comprise the internalising score); (e) aggressive behaviour and (f) attention problems syndrome (e–f comprise the externalising score); and (g) a sleep problem score and (h) other problems (g–h comprise the score for other problems). The total score was calculated by summing all eight subscales. The study also utilised this scale in determining the categories of the study subjects: children with *t*-scores ≤59 were classified as not having any behavioural issues, while *t*-scores ≥60 but ≤64 were classified as at risk and *t*-scores ≥65 considered as clinical.^2^ CBCL has been found to be a valid and reliable measure for childhood behaviour concerns, with strong internal consistency; in the current study, there was excellent internal consistency for the CBCL externalising scale (Cronbach’s alpha = 0.90) and CBCL total (Cronbach’s alpha = 0.93), while it was slightly lower and yet robust for CBCL internalising scale (Cronbach’s alpha = 0.86). For the purposes of this study, CBCL total, internalising and externalising scores were used as outcome measures.

#### ADOS-2

ADOS-2 is an assessor-led, semi-structured, standardised diagnostic observational assessment used to confirm autism diagnosis.^16^ It contains specific developmental and language level-dependent modules (modules 1–3) that examine autistic traits in two domains – restricted and repetitive behaviours (RRB) and social affect. To account for differences between modules, scores for RRB and social affect domains, as well as total score were converted into calibrated severity scores based on previous validation studies, in which comparisons with the severity of autism symptomatology across modules can be carried out; a higher score indicates greater severity.^17^

#### SCQ

The Social Communication Questionnaire (SCQ) is a self-reported, 40-item questionnaire that measures autistic traits in the social communication domain.^18^ The SCQ is completed by parents with either a ‘yes’ or ‘no’ response covering reciprocal social interaction, language and communication and repetitive and stereotyped patterns of behaviour, with a higher score indicating greater severity. The current study showed good (Cronbach’s alpha = 0.89) internal consistency between items.

#### RBS-R

Repetitive Behaviour Scale – Revised (RBS-R) is a 43-item, parent-completed questionnaire that measures repetitive behaviours in autistic children, adolescents and adults.^19^ It is divided into six subscales of behaviours: stereotyped, self-injurious, compulsive, ritualistic, sameness and restricted behaviour. It is scored on a 3-point scale, with 0 indicating not present and 3 indicating a severe concern. The total score is computed, with a higher score indicating greater severity. For the current study, there was excellent (Cronbach’s alpha = 0.94) internal consistency between items.

#### MSEL

The Mullen Scales of Early Learning (MSEL) assesses children’s development across key domains including language, motor, perceptual abilities, cognitive ability and motor development.^20^ The assessment provides four subscales (visual reception, fine motor, receptive and expressive language) and a standardised and age-equivalent overall early learning composite. In the current study, raw scores of the first three domains and a corresponding age-equivalent score were obtained. A standardised developmental quotient ((age equivalent/chronological age) × 100) was calculated for both non-verbal (mean fine motor and visual reception) and verbal (mean receptive and expressive language) domains,^21^ where a higher score indicates better cognitive functioning.

#### VABS-II

The Vineland Adaptive Behaviour Scales, second edition (VABS-II) parent/caregiver rating form assesses an individual’s adaptive functioning in five domains: communication, daily living skills, socialisation, motor skills and maladaptive behaviour.^22^ All items on VABS-II are scored as either 0 (behaviour never performed/never occurs without help), 1 (behaviour is sometimes performed without help or reminders) or 2 (behaviour usually occurs without help). Adaptive behaviour composite scores were computed by summing the scores from the 4 domains (except maladaptive behaviour); these ranged from 20 to 160, where a higher score indicates better adaptive functioning.

#### Sociodemographic factors

Sociodemographic items included the child’s age (in years) and gender (male, female); parent’s age (in years); culturally and linguistically diverse background (CALD) status (refers to people born in non-English-speaking countries and/or who do not speak English at home^23^) (yes, no); sibling’s autism diagnosis (no, yes); caregiver’s disability status (no, yes); carer’s level of education (primary/secondary, postgraduate/tertiary); occupation (professional/paraprofessional, other labour); and annual family income (in Australian dollars (AUD), <AUD40 000, AUD40 001–85 000, AUD85 001–115 000, >AUD115 000).

### Data analysis

The characteristics of the sample were analysed using descriptive statistics, and are presented as mean and standard deviations for continuous measures and as frequency counts with percentages for categorical measures. Given the missing data in the sample, multiple imputation using chained equations was applied^24^ and variables with <50% missingness on the CBCL were imputed. Multiple imputation makes repeated draws from the model of distribution of variables and provides valid values using other available information from the data-set.^25^ Incomplete variables were imputed under fully conditional specification and combined using Rubin’s rules. The scores for each subscale (e.g. withdrawn subscale, internalising scale or total) were recalculated using imputed estimates. These recalculated scores were also compared against the non-imputed subscale scores to ensure that there were no significant differences that might impact the mean.

A univariate linear regression was first carried out to determine the independent association of each independent variable against the outcome variable. Pearson’s correlation analysis was conducted to determine any significant correlation between each of the clinical indicators and sociodemographic variables. With the large number of sociodemographic, cultural and socioeconomic variables, only variables with a *P*-value ≤0.20 in the univariate regression analysis, and those not significantly correlated, were entered into the multivariable models. This approach in statistical modelling helps to reduce potential overfitting and noise from other highly non-significant variables in the univariate model, as well as improving model interpretability. This less stringent cut-off (e.g. *P* ≤ 0.20 rather than the conventional 0.05) helps in avoiding the exclusion of potentially important predictors that may become significant when adjusting for confounders in the multivariate analysis.^26^

A multivariable regression analysis model was carried out to examine the association between autistic and child features, including social communication differences, repetitive behaviours, cognitive level, adaptive functioning (predictors) and mental health challenges (CBCL internalising, externalising and total score) as outcome variables adjusted for sociodemographic variables. Additionally, we also examined associations among key sociodemographic variables against the mental health challenges. Findings from the regression models are reported as standardised beta coefficient (*β*) with 95% confidence interval and *P*-value (*P*). All statistical analyses were performed using Statistical Package for Social Sciences (SPSS) v.28 (SPSS for macOS, SPSS Inc., Chicago, Illinois, USA, https://www.ibm.com/products/spss) and R language v.3.6.1 within RStudio IDE (for macOS, R Foundation for Statistical Computing, Vienna, Austria, https://cran.r-project.org).

## Results

### Descriptive characteristics of the sample

The descriptive characteristics of the children and their families are outlined in Table 1. The average age of the participants was 3.47 ± 0.87 years, and 80% were male. Around a third of the total sample had an autistic sibling and 42.1% of the total sample were from a CALD background.

**Table 1:** Baseline demographic characteristics (*N* = 760) of autistic children and their parents/caregivers

Variables	*n*	%
Child’s age in years, mean (s.d.)	760	3.47 (0.87)
Mother’s age in years, mean (s.d.)	760	34.40 (5.21)
Father’s age in years, mean (s.d.)	760	37.41 (6.48)
Child’s gender		
Male	607	80.0
Female	152	20.0
CALD status		
Non-CALD	405	57.9
CALD	295	42.1
Sibling’s ASD diagnosis		
No	369	66.0
Yes	190	34.0
Caregiver’s disability		
No	480	83.2
Yes	97	16.8
Primary carer’s education level		
Primary/secondary	202	29.1
Postgraduate/tertiary	493	70.9
Secondary carer’s education level		
Primary/secondary	214	33.6
Postgraduate/tertiary	423	66.4
Primary carer’s occupation		
Professional/paraprofessional	188	27.6
Other labour	492	72.4
Secondary carer’s occupation		
Professional/paraprofessional	297	46.8
Other labour	338	53.2
Annual family income		
<AUD40 000	154	26.6
AUD40 001–85 000	191	32.9
AUD85 001–115 000	115	19.8
>AUD115 000	120	20.7

CALD, culturally and linguistically diverse; ASD, autism spectrum disorder; AUD, Australian dollar.

The baseline characteristics of autistic features, cognitive and adaptive functioning, as well as mental health issues, are detailed in Tables 2 and 3. Regarding mental health difficulties, 59% reported internalising concerns, 21.0% reported externalising behavioural challenges and 58.7% exhibited overall behavioural difficulties. Out of the seven CBCL subscales, three behavioural problems (withdrawn problem, attention problems and somatic complaints) had more children in the at-clinical group than the average group.

**Table 2:** Behavioural and cognitive measures of autistic children

Variables	*N*	Mean (s.d.)
Child Behavioural Checklist (CBCL)		
CBCL internalising scores	760	64.01 (7.55)
CBCL externalising scores	760	59.72 (9.22)
CBCL total problem scores	760	64.79 (8.89)
Emotionally reactive subscale	760	57.99 (6.11)
Anxious/depressed subscale	760	57.92 (7.50)
Somatic complaints subscale	760	61.06 (7.30)
Withdrawn subscale	760	72.78 (8.48)
Sleep problems subscale	760	59.43 (8.61)
Attention problems subscale	760	63.54 (7.28)
Aggressive behaviour subscale	760	59.38 (8.74)
Autism Diagnostic Observation Schedule (ADOS-2) assessment		
ADOS-2 social affect	760	13.60 (3.97)
ADOS-2 restricted and repetitive behaviour	760	4.40 (1.95)
ADOS-2 calibrated severity scale	760	7.22 (1.73)
Social communication problems score	760	19.13 (5.59)
Repetitive behaviours scale-revised score	760	33.60 (17.34)
Mullen non-verbal developmental quotient	760	60.42 (22.97)
Mullen verbal developmental quotient	760	46.81 (26.69)
Vineland adaptive behaviour composite score	500	68.02 (12.57)

**Table 3:** Frequency of autistic children in average and at-risk/clinical groups for total Child Behavioural Checklist and subscales (*N* = 760)

Total and subscales	Average (%)	At-risk (%)	Clinical (%)
Total	23.7	17.6	58.7
Internalising	20.9	20.1	59.0
Externalising	47.8	31.2	21.0
Emotional reactive	66.3	16.7	17.0
Anxiety/depression	68.9	12.2	18.9
Somatic complaints	39.6	15.7	44.7
Withdrawn	8.6	7.6	83.8
Sleep problems	59.7	23.6	16.7
Attention problems	30.3	24.2	45.5
Aggressive behaviour	59.3	22.1	18.6

### Associations among autistic features, cognitive and adaptive functioning and mental health difficulties

Findings from the multivariable linear regression models showing associations between mental health difficulties and autistic traits are shown in Table 4.

**Table 4:** Multi-level linear regression analysis with behavioural, communication, cognitive and adaptive traits associated with CBCL internalising and externalising problems (unadjusted and adjusted models, adjusted for sociodemographic covariates)

Variable	CBCL internalising problem		CBCL externalising problem		CBCL total	
	Unadjusted model	Adjusted model	Unadjusted model	Adjusted model	Unadjusted model	Adjusted model
	*β* (95% CI)	*β* (95% CI)	*β* (95% CI)	*β* (95% CI)	*β* (95% CI)	*β* (95% CI)
Autism severity (ADOS-2 CSS)	0.16 (−0.15, 0.47)	0.44 (0.02, 0.86)*	−0.40 (−0.78, −0.02)*	0.03 (−0.49, 0.55)	−0.04 (−0.41, 0.33)	0.33 (−0.16, 0.81)
Social communication deficit (SCQ)	0.51 (0.42, 0.60)*	0.54 (0.42, 0.66)*	0.38 (0.27, 0.50)*	0.37 (0.22, 0.53)*	0.57 (0.46, 0.67)*	0.56 (0.42, 0.70)*
Repetitive and/or restrictive behaviour (RBS-R)	0.26 (0.24, 0.29)*	0.27 (0.23, 0.30)*	0.29 (0.25, 0.32)*	0.30 (0.26, 0.34)*	0.34 (0.31, 0.36)*	0.34 (0.30, 0.37)*
MSEL non-verbal developmental functioning	−0.03 (−0.05, −0.01)*	−0.02 (−0.06, 0.01)	0.03 (0.01, 0.06)*	0.02 (−0.02, 0.06)	−0.01 (−0.04, 0.13)	−0.03 (−0.07, 0.01)
MSEL verbal developmental functioning	−0.03 (−0.05, −0.01)*	−0.04 (−0.06, −0.01)*	0.03 (0.01, 0.06)*	0.03 (−0.01, 0.06)	−0.02 (−0.04, 0.01)	−0.02 (−0.06, 0.01)
Vineland adaptive functioning	−0.17 (−0.22, −0.11)*	−0.17 (−0.26, −0.09)*	−0.07 (−0.14, −0.01)*	−0.10 (−0.19, −0.01)*	−0.16 (−0.23, −0.10)*	−0.20 (−0.29, −0.11)*

CBCL, Child Behavioural Checklist; ADOS-2, Autism Diagnostic Observation Schedule − Second Edition; CSS, calibrated severity score; SCQ, Social Communication Questionnaire; RBS-R, Repetitive Behaviour Scale − Revised; MSEL, Mullen Scale of Early Learning. *P < 0.05.

Consistent with the univariate models, findings from the multivariable models showed that higher scores on both social communication difficulties and repetitive and/or restricted behaviours were significantly associated with higher internalising (*β* = 0.54, 95% CI 0.42, 0.66), externalising (*β* = 0.37, 95% CI 0.22, 0.53) and total problem scores (*β* = 0.56, 95% CI 0.42, 0.70). On the other hand, higher adaptive functioning was associated with lower internalising (*β* = −0.17, 95% CI −0.26, −0.09), externalising (*β* = −0.10, 95% CI −0.19, −0.01) and total problem scores (*β* = −0.20, 95% CI −0.29, −0.11). Furthermore, greater severity of autistic symptoms was significantly associated with higher internalising behaviours (*β* = 0.44, 95% CI 0.02, 0.86) but not with externalising or total problems. Additionally, higher scores on verbal developmental functioning were significantly linked to lower internalising behavioural problems (*β* = −0.04, 95% CI −0.06, −0.01).

### Associations among sociodemographic and sociocultural factors and mental health difficulties

The associations among mental health challenges and sociodemographic factors were also examined (Table 5). Findings showed that increase in children’s age was associated with higher internalising (*β* = 1.56, 95% CI 0.69, 2.44), externalising (*β* = 1.50, 95% CI 0.43, 2.57) and total problems (*β* = 1.59, 95% CI 0.58, 2.59). Compared with males, females reported significantly higher internalising problems (*β* = 2.17, 95% CI 0.30, 2.44). Furthermore, compared with siblings without an autistic diagnosis, children with an autistic sibling reported significantly higher internalising (*β* = 2.50, 95% CI 0.96, 4.04), externalising (*β* = 3.77, 95% CI 1.89, 5.66) and total problems (*β* = 3.97, 95% CI 2.20, 5.74).

**Table 5:** Multi-level linear regression analysis showing sociodemographic factors associated with CBCL internalising, externalising and total problem (unadjusted and adjusted models, adjusted for behavioural, communication, cognitive and adaptive traits)

Variable	CBCL internalising		CBCL externalising		CBCL total	
	Unadjusted model	Adjusted model	Unadjusted model	Adjusted model	Unadjusted model	Adjusted model
	*β* (95% CI)	*β* (95% CI)	*β* (95% CI)	*β* (95% CI)	*β* (95% CI)	*β* (95% CI)
Child’s age	1.52 (0.91, 2.13)*	1.56 (0.69, 2.44)*	1.38 (0.63, 2.13)*	1.50 (0.43, 2.57)*	1.48 (0.76, 2.20)*	1.59 (0.58, 2.59)*
Child’s gender						
Male	Reference	Reference	Reference	Reference	Reference	Reference
Female	1.69 (0.36, 3.04)*	2.17 (0.30, 2.44)*	0.80 (−0.85, 2.44)	0.54 (−1.75, 2.83)	1.71 (0.13, 3.29)*	2.00 (−0.14, 4.15)
CALD background status						
Non-CALD	Reference	Reference	Reference	Reference	Reference	Reference
CALD	−0.10 (−1.26, 1.06)	0.01 (−1.51, 1.51)	−1.87 (−3.27, −0.46)*	−1.96 (−3.81, −0.11)*	−1.07 (−2.43, 0.29)	−0.83 (−2.57, 0.91)
Sibling ASD						
No	Reference	Reference	Reference	Reference	Reference	Reference
Yes	1.62 (0.25, 2.99)*	2.50 (0.96, 4.04)*	3.07 (1.39, 4.74)*	3.77 (1.89, 5.66)*	3.02 (1.41, 4.63)*	3.97 (2.20, 5.74)*
Mother’s age	−0.09 (−0.19, 0.01)	−0.06 (−0.21, 0.08)		−0.09 (−0.27, 0.08)	−0.13 (−0.25, −0.01)*	−0.08 (−0.24, 0.08)
Primary carer education						
Primary/secondary	Reference	Reference	Reference	Reference	Reference	Reference
Postgraduate/tertiary	−1.93 (−3.19, −0.67)*	−1.19 (−2.85, 0.46)	−0.10 (−0.22, 0.03)	−1.89 (−3.92, 0.14)	−2.79 (−4.27, −1.31)*	−1.87 (−3.77, −0.01)*
Annual income						
<AUD40 000	Reference	Reference	Reference	Reference	Reference	Reference
AUD40 001–85 000	−1.12 (−2.69, 0.47)	−0.45 (−2.36, 1.45)	−1.20 (−3.15, 0.75)	−0.19 (−2.52, 2.15)	−1.87 (−3.71, −0.03)*	−0.88 (−3.06, 1.31)
AUD85 001–115 000	−3.29 (−5.09, −1.49)*	−1.95 (−4.13, 0.23)	−2.84 (−5.07, −0.62)*	−1.19 (−3.86, 1.49)	−4.30 (−6.39, −2.21)*	−2.41 (−4.92, 0.09)
>AUD115 000	−4.35 (−6.13, −2.57)*	−2.74 (−4.92, −0.56)*	−2.37 (−4.56, −0.17)*	−1.07 (−3.75, 1.60)	−4.56 (−6.63, −2.49)*	−2.78 (−5.29, −0.27)*

CBCL, Child Behavioural Checklist; CALD, culturally and linguistically diverse; ASD, autism spectrum disorder; AUD, Australian dollar. *P < 0.05.

Interestingly, children from a CALD background were reported as having significantly lower externalising problems (*β* = −1.96, 95% CI −3.81, −0.11). Furthermore, higher levels of parental education were associated with significantly lower total problems (*β* = −1.87, 95% CI −3.77, −0.01), and annual family income was also protective against both internalising (*β* = −2.74, 95% CI −4.92, −0.56) and total problems (*β* = −2.78, 95% CI −5.29, −0.27).

## Discussion

### Summary of findings

This study aimed to determine the association between behavioural and mental health challenges in autistic preschoolers and their association with autistic traits, cognitive level, adaptive functioning and sociodemographic factors. The findings of this study provide useful insights into the high prevalence of mental health difficulties, via CBCL internalising, externalising and total problem scores, among autistic preschool children. The study found a significant association between autistic traits and mental health difficulties. Additionally, sociodemographic factors such as being older, female and having a sibling with autism, were linked to a higher risk of mental health challenges, while a CALD background, higher parental education level and greater family income appeared to offer a protective effect.

### Prevalence of mental health challenges

This study found that behavioural concerns are common among autistic preschoolers, occurring in as many as three-quarters of children. This is consistent with the findings of Guerrera et al,^2^ who found that around 50% of autistic children exhibited CBCL total scores in the at-risk or clinical range. Because that previous study looked at an older sample (ages 2.6–17.8 years, mean 5.45 years), it is possible that the relatively lower rates seen were a function of the decline in behavioural concerns as children age. Furthermore, it has been shown that externalising behaviours may be more common at lower severities at a younger age,^2^ while our findings suggest higher rates of internalising behaviours in the preschool age group. While this finding of higher internalising than externalising behaviours is also consistent with that of Guerrera et al,^2^ the overall rate for both internalising and externalising behaviours in this study was higher. This might be due to the fact that the children in the current study were attending a specialised early intervention programme for autistic children, whereas those in the study of Guerrera et al were attending neuropsychiatry out-patient units for clinical assessments and rehabilitative follow-ups.

The high prevalence of emotion regulation issues in autistic preschoolers, and their limited capacity for self-regulation to help control their emotion, attention and activities, may manifest as either internalising or externalising behaviours.^27^ Also, it is possible that in the early years the fears or worries (internalising behaviours) that they experience will manifest more as externalising behaviours, based on family and contextual factors such as parental and family functioning.^27^ Given the heterogeneity in the development of internalising and externalising behaviours in autistic preschool children, and the limited knowledge about the development of these behaviours in either isolation or combination,^2^ further research into autistic preschoolers is warranted. Furthermore, our findings are also in keeping with previous studies that have reported higher prevalences of withdrawal,^28,29^ attention difficulties^29^ and somatic complaints^30^ relative to other subscales.

### Association between mental health difficulties and autistic traits

The finding that autistic children with high repetitive and/or restricted behaviours had more internalising and externalising behaviours is consistent with the existing literature.^31^ This might be explained by the fact that, when autistic children are anxious, they may attempt to control their environment to overcome further stimulation, uncertainty and consequent increase in anxiety by using RRBs such as restricted interests and insistence on sameness as a coping behaviour.^31^ Furthermore, we found that children with greater social communication differences exhibited higher internalising and externalising behaviours, consistent with findings from prior studies.^29,30^ Such differences may hinder their ability to interpret others’ emotions, express their own feelings and assess social responses,^32^ making social interactions seem unpredictable. This can evoke fear (internalising) or frustration (externalising), creating a cycle of awkward peer interactions, social withdrawal and heightened anxiety or, conversely, tantrums and aggression.^33^

Our study’s finding that higher levels of adaptive functioning and verbal developmental functioning are linked to lower internalising and externalising problems aligns with established developmental and clinical theories.^34^ Enhanced adaptive skills may serve as protective factors against psychopathology by facilitating more effective communication, reducing frustration and enabling positive social interactions with peers and caregivers. These skills increase opportunities for meaningful participation in daily activities and social contexts which, in turn, supports emotional regulation and behavioural adjustment.^35^ Verbal developmental functioning further contributes by providing children with the tools to express needs and emotions verbally rather than through maladaptive behaviours, thereby reducing internal distress and externalising reactions.^36^ Together, these capacities may help buffer children from developing significant mental health difficulties by fostering adaptive coping strategies and resilience within social environments.

### Association between mental health challenges and sociodemographic covariates

Besides autism severity and autistic features, several sociodemographic and sociocultural factors were found to be significantly associated with mental health challenges. Older-aged children exhibited more internalising, externalising and total behaviours than younger children, which might be related to the difficulty in adjusting their behaviours to the changing social and environmental demands, which was greater in older autistic children than in younger ones.^2^ Gender played a key role, whereby girls reported a higher likelihood of internalising behaviours than boys, which may be linked to the differences in developmental mechanisms and trajectories in autistic females and males.^37^ Consistent with previous research, autistic children with an autistic sibling in our sample showed higher internalising, externalising and total behaviour problem scores. This may reflect a combination of shared genetic liability for emotional and behavioural difficulties, as well as environmental stressors within families raising multiple autistic children.^38^

Interestingly, children from CALD backgrounds were reported to have significantly lower externalising problems, suggesting that cultural norms emphasising behavioural restraint, respect for authority and strong family cohesion may act as protective factors against overt behavioural difficulties.^39^ Additionally, the protective role of parental education and family income in behaviours of concern is consistent with prior evidence that higher education and higher family income may equip parents with greater knowledge, problem-solving skills and access to resources that support child emotional and behavioural regulation.^40,41^

### Strengths, limitations and directions for future research

This study has several strengths and limitations. Among the key strengths is the inclusion of its large and well-characterised clinical sample drawn from six Australian ASELCCs, which enhances the relevance of findings to children accessing specialised early intervention services. Furthermore, the use of validated measures for autistic traits, cognitive and adaptive functioning and mental health difficulties, combined with multivariable regression analyses, strengthens the robustness of the observed associations. However, we also note several limitations. Due to the nature of the cross-sectional data, causal inferences and developmental trajectories could not be examined. The sample’s restriction to ASELCC attendees may have limited generalisability to the wider autistic population, potentially introducing selection bias. Additionally, reliance on parent-reported instruments could have introduced reporting bias and may not fully capture the complex mental health profiles seen in autism. Future research should aim to build on these findings by employing longitudinal designs to elucidate the developmental trajectories and causal pathways linking autistic traits, adaptive functioning and mental health outcomes. Additionally, expansion of research to include broader community samples beyond specialised early learning centres will enhance generalisability.

### Implications for policy and practice

The findings of this study underscore the critical importance of early and comprehensive assessment of, and support for, autistic features, adaptive functioning, verbal skills and mental health difficulties among preschool children. Given the protective role of adaptive and verbal developmental functioning, interventions that enhance communication and daily living skills may be particularly beneficial in mitigating internalising and externalising problems. Furthermore, the inextricable link between sociodemographic and sociocultural factors and mental health challenges underscores the need for family-centred, culturally sensitive approaches and equitable access to resources, ensuring that families from diverse backgrounds receive appropriate and tailored support.

## Data Availability

The data-sets generated and/or analysed during the current study are not publicly available due to data governance arrangements. However, aggregate de-identified data may be available from the corresponding author on reasonable request.

## References

[1] American Psychiatric Association. Diagnostic and Statistical Manual of Mental Disorders. APA, 2013.

[2] Guerrera S , Menghini D , Napoli E , Di Vara S , Valeri G , Vicari S. Assessment of psychopathological comorbidities in children and adolescents with autism spectrum disorder using the Child Behavior Checklist. Front Psychiatry 2019; 10: 535. 31404318 10.3389/fpsyt.2019.00535 PMC6676343

[3] Fung LK , Mahajan R , Nozzolillo A , Bernal P , Krasner A , Jo B , et al. Pharmacologic treatment of severe irritability and problem behaviors in autism: a systematic review and meta-analysis. Pediatrics 2016; 137: S124–35. 26908468 10.1542/peds.2015-2851K

[4] Kanne SM , Mazurek MO. Aggression in children and adolescents with ASD: prevalence and risk factors. J Autism Dev Disord 2011; 41: 926–37. 20960041 10.1007/s10803-010-1118-4

[5] Nazeer A. Psychopharmacology of autistic spectrum disorders in children and adolescents. Pediatr Clin 2011; 58: 85–97. 10.1016/j.pcl.2010.10.011 21281850

[6] McGuire K , Fung LK , Hagopian L , Vasa RA , Mahajan R , Bernal P , et al. Irritability and problem behavior in autism spectrum disorder: a practice pathway for pediatric primary care. Pediatrics 2016; 137: S136–48. 26908469 10.1542/peds.2015-2851L

[7] Lugnegård T , Hallerbäck MU , Gillberg C. Psychiatric comorbidity in young adults with a clinical diagnosis of Asperger syndrome. Res Dev Disabil 2011; 32: 1910–7. 21515028 10.1016/j.ridd.2011.03.025

[8] Sterling L , Dawson G , Estes A , Greenson J. Characteristics associated with presence of depressive symptoms in adults with autism spectrum disorder. J Autism Dev Disord 2008; 38: 1011–8. 17975722 10.1007/s10803-007-0477-y

[9] Chan N , Fenning RM , Neece CL. Prevalence and phenomenology of anxiety in preschool-aged children with autism spectrum disorder. Res Child Adolesc Psychopathol 2023; 51: 33–45. 36048376 10.1007/s10802-022-00964-8

[10] Kaat AJ , Lecavalier L , Aman MG. Validity of the aberrant behavior checklist in children with autism spectrum disorder. J Autism Dev Disord 2014; 44: 1103–16. 24165702 10.1007/s10803-013-1970-0

[11] Bacherini A , Igliozzi R , Cagiano R , Mancini A , Tancredi R , Muratori F , et al. Behavioral and emotional problems of toddlers with autism spectrum disorder: effects of parents’ sociocultural level and individual factors. Res Dev Disabil 2021; 119: 104106. 34656889 10.1016/j.ridd.2021.104106

[12] Rescorla L , Kim YA , Oh KJ. Screening for ASD with the Korean CBCL/1½–5. J Autism Dev Disord 2015; 45: 4039–50. 25239178 10.1007/s10803-014-2255-y

[13] Von Elm E , Altman DG , Egger M , Pocock SJ , Gøtzsche PC , Vandenbroucke JP , et al. The Strengthening the Reporting of Observational Studies in Epidemiology (STROBE) statement: guidelines for reporting observational studies. Int J Surg 2014; 12: 1495–9.25046131

[14] Masi A , Dissanayake C , Alach T , Cameron K , Fordyce K , Frost G , et al. Clinical outcomes and associated predictors of early intervention in autism spectrum disorder: a study protocol. BMJ Open 2021; 11: e047290. 10.1136/bmjopen-2020-047290 PMC8354249 34373300

[15] Achenbach TM , Rescorla LA. Manual for the ASEBA Preschool Forms and Profiles. University of Vermont, Research Center for Children, Youth, & Families, 2000.

[16] Lord C , Rutter M , DiLavore P , Risi S , Gotham K , Bishop S. Manual for the Autism Diagnostic Observation Schedule: ADOS-2. Western Psychological Services, 2012.

[17] Hus V , Gotham K , Lord C. Standardizing ADOS domain scores: separating severity of social affect and restricted and repetitive behaviors. J Autism Dev Disord 2014; 44: 2400–12. 23143131 10.1007/s10803-012-1719-1 PMC3612387

[18] Lord C , Michael R , Rutter M. Social Communication Questionnaire (SCQ). Western Psychological Services, 2003.

[19] Lam KS , Aman MG. The Repetitive Behavior Scale-Revised: independent validation in individuals with autism spectrum disorders. J Autism Dev Disord 2007; 37: 855–66. 17048092 10.1007/s10803-006-0213-z

[20] Mullen EM. Mullen Scales of Early Learning, AGS ed. American Guidance Service Inc, 1995.

[21] Messinger D , Young GS , Ozonoff S , Dobkins K , Carter A , Zwaigenbaum L , et al. Beyond autism: a baby siblings research consortium study of high-risk children at three years of age. J Am Acad Child Adolesc Psychiatry 2013; 52: 300–8. 23452686 10.1016/j.jaac.2012.12.011 PMC3625370

[22] Sparrow SS , Balla D , Cicchetti DV , Doll EA. Vineland II: Vineland Adaptative Behavior Scales: Survey Forms Manual: A Revision of the Vineland Social Maturity Scale. Pearson, 2005.

[23] Pham TTL , Berecki-Gisolf J , Clapperton A , O’Brien KS , Liu S , Gibson K. Definitions of culturally and linguistically diverse (CALD): a literature review of epidemiological research in Australia. Int J Environ Res Public Health 2021; 18: 737. 33467144 10.3390/ijerph18020737 PMC7830035

[24] Azur MJ , Stuart EA , Frangakis C , Leaf PJ. Multiple imputation by chained equations: what is it and how does it work? Int J Methods Psychiatr Res 2011; 20: 40–9. 21499542 10.1002/mpr.329 PMC3074241

[25] Rubin DB. Multiple imputation after 18+ years. J Am Stat Assoc 1996; 91: 473–89.

[26] Chowdhury MZI , Turin TC. Variable selection strategies and its importance in clinical prediction modelling. Fam Med Community Health 2020; 8: e000262. 32148735 10.1136/fmch-2019-000262 PMC7032893

[27] Nuske HJ , Hedley D , Tseng CH , Begeer S , Dissanayake C. Emotion regulation strategies in preschoolers with autism: associations with parent quality of life and family functioning. J Autism Dev Disord 2018; 48: 1287–300. 29192379 10.1007/s10803-017-3391-y

[28] Ooi YP , Rescorla L , Ang RP , Woo B , Fung DS. Identification of autism spectrum disorders using the child behavior checklist in Singapore. J Autism Dev Disord 2011; 41: 1147–56. 20405192 10.1007/s10803-010-1015-x

[29] Kim JY , Ha EH. Cluster analysis of the Child Behavior Checklist 1.5–5 for preschool children diagnosed with a mental disorder. Psychol Rep 2020; 123: 1403–24. 31046625 10.1177/0033294119844980

[30] Leader G , Flynn C , O’Rourke N , Coyne R , Caher A , Mannion A. Comorbid psychopathology, challenging behavior, sensory issues, adaptive behavior and quality of life in children and adolescents with autism spectrum disorder. Dev Neurorehabil 2021; 24: 397–407. 33706637 10.1080/17518423.2021.1898058

[31] Rodgers J , Glod M , Connolly B , McConachie H. The relationship between anxiety and repetitive behaviours in autism spectrum disorder. J Autism Dev Disord 2012; 42: 2404–9. 22527704 10.1007/s10803-012-1531-y

[32] Mazefsky CA , Herrington J , Siegel M , Scarpa A , Maddox BB , Scahill L , et al. The role of emotion regulation in autism spectrum disorder. J Am Acad Child Adolesc Psychiatry 2013; 52: 679–88. 23800481 10.1016/j.jaac.2013.05.006 PMC3719386

[33] Li B , Bos MG , Stockmann L , Rieffe C. Emotional functioning and the development of internalizing and externalizing problems in young boys with and without autism spectrum disorder. Autism 2020; 24: 200–10. 31549858 10.1177/1362361319874644 PMC6927076

[34] Phillips SJP. From Neurons to Neighborhoods: The Science of Early Childhood Development. National Academy Press, 2000. 25077268

[35] Donoso J , Rattray F , De Bildt A , Tillmann J , Williams P , Absoud M , et al. Association of cognitive and adaptive skills with internalizing and externalizing problems in autistic children and adolescents. Autism Res 2024; 17: 596–609. 38031634 10.1002/aur.3056

[36] Chow JC , Ekholm E , Coleman H. Does oral language underpin the development of later behavior problems? A longitudinal meta-analysis. School Psychol Quart 2018; 33: 337–49 10.1037/spq0000255 29792491

[37] Stephenson KG , Norris M , Butter EM. Sex-based differences in autism symptoms in a large, clinically-referred sample of preschool-aged children with ASD. J Autism Dev Disord 2023; 53: 624–32. 33459916 10.1007/s10803-020-04836-2

[38] Sandin S , Lichtenstein P , Kuja-Halkola R , Hultman C , Larsson H , Reichenberg A. The heritability of autism spectrum disorder. JAMA 2017; 318: 1182–4. 28973605 10.1001/jama.2017.12141 PMC5818813

[39] Yap MB , Cheong TW , Zaravinos-Tsakos F , Lubman DI , Jorm AF. Modifiable parenting factors associated with adolescent alcohol misuse: a systematic review and meta-analysis of longitudinal studies. Addiction 2017; 112: 1142–62. 28178373 10.1111/add.13785

[40] Fuller-Thomson E , Sawyer JL. Is the cluster risk model of parental adversities better than the cumulative risk model as an indicator of childhood physical abuse? Findings from two representative community surveys. Child Care Health Dev 2014; 40: 124–33. 23278274 10.1111/cch.12024

[41] Kohen D , Guèvremont A. Income disparities in preschool outcomes and the role of family, child, and parenting factors. Early Child Dev Care 2014; 184: 266–92.

